# Machine learning derived serum creatinine trajectories in acute kidney injury in critically ill patients with sepsis

**DOI:** 10.1186/s13054-024-04935-x

**Published:** 2024-05-10

**Authors:** Kullaya Takkavatakarn, Wonsuk Oh, Lili Chan, Ira Hofer, Khaled Shawwa, Monica Kraft, Neomi Shah, Roopa Kohli-Seth, Girish N. Nadkarni, Ankit Sakhuja

**Affiliations:** 1 Division of Nephrology, Department of Medicine, Icahn School of Medicine at Mount Sinai, New York, NY USA; 2 Division of Nephrology, Department of Medicine, King Chulalongkorn Memorial Hospital, Chulalongkorn University, Bangkok, Thailand; 3 The Charles Bronfman Institute for Personalized Medicine, Icahn School of Medicine at Mount Sinai, New York, NY USA; 4 Division of Data Driven and Digital Medicine, Department of Medicine, Icahn School of Medicine at Mount Sinai, New York, NY USA; 5 Department of Anesthesiology, Icahn School of Medicine at Mount Sinai, New York, NY USA; 6 Division of Nephrology, Department of Medicine, West Virginia University, Morgantown, WV USA; 7 Division of Pulmonary, Critical Care and Sleep Medicine, Department of Medicine, Icahn School of Medicine at Mount Sinai, New York, NY USA; 8 Institute for Critical Care Medicine, Icahn School of Medicine at Mount Sinai, New York, NY USA

**Keywords:** Acute kidney injury, Creatinine trajectory, Critical care, Sepsis

## Abstract

**Background:**

Current classification for acute kidney injury (AKI) in critically ill patients with sepsis relies only on its severity-measured by maximum creatinine which overlooks inherent complexities and longitudinal evaluation of this heterogenous syndrome. The role of classification of AKI based on early creatinine trajectories is unclear.

**Methods:**

This retrospective study identified patients with Sepsis-3 who developed AKI within 48-h of intensive care unit admission using Medical Information Mart for Intensive Care-IV database. We used latent class mixed modelling to identify early creatinine trajectory-based classes of AKI in critically ill patients with sepsis. Our primary outcome was development of acute kidney disease (AKD). Secondary outcomes were composite of AKD or all-cause in-hospital mortality by day 7, and AKD or all-cause in-hospital mortality by hospital discharge. We used multivariable regression to assess impact of creatinine trajectory-based classification on outcomes, and eICU database for external validation.

**Results:**

Among 4197 patients with AKI in critically ill patients with sepsis, we identified eight creatinine trajectory-based classes with distinct characteristics. Compared to the class with transient AKI, the class that showed severe AKI with mild improvement but persistence had highest adjusted risks for developing AKD (OR 5.16; 95% CI 2.87–9.24) and composite 7-day outcome (HR 4.51; 95% CI 2.69–7.56). The class that demonstrated late mild AKI with persistence and worsening had highest risks for developing composite hospital discharge outcome (HR 2.04; 95% CI 1.41–2.94). These associations were similar on external validation.

**Conclusions:**

These 8 classes of AKI in critically ill patients with sepsis, stratified by early creatinine trajectories, were good predictors for key outcomes in patients with AKI in critically ill patients with sepsis independent of their AKI staging.

**Supplementary Information:**

The online version contains supplementary material available at 10.1186/s13054-024-04935-x.

## Background

Acute kidney injury (AKI) is a common complication in up to 60% of patients with critical illness and is associated with high morbidity and mortality [1]. Sepsis is the most common cause of AKI among critically ill patients [2]. Sepsis patients with AKI, have mortality rates six to eight folds higher than those without AKI [3, 4]. AKI in critically ill patients with sepsis is also associated with a higher risk of worsening kidney function including acute kidney disease (AKD), chronic kidney disease (CKD) and end-stage kidney disease (ESKD) [5–7]. The risks of these complications increase with increasing severity of AKI as defined by Kidney Disease Improving Global Outcomes (KDIGO) AKI staging [2, 7, 8].

Recent evidence has shown that AKI in sepsis patients is a heterogenous syndrome [9] with multiple mechanisms. These include inflammation, mitochondrial dysfunction, metabolic reprogramming, and microcirculatory dysfunction [10]. Additionally, sepsis associated factors including use of nephrotoxic medications or associated complications such as volume overload can further contribute to AKI in patients with sepsis [11, 12]. Therefore, relying on the assessment of AKI severity and patient prognosis solely based on the maximum changes in serum creatinine, as current KDIGO AKI staging does, without considering the longitudinal characteristics of serum creatinine changes, is an oversimplification, as it overlooks the complexities of this heterogenous syndrome.

The trajectory of serum creatinine, identified by the trend of percent change in serum creatinine over time, has been shown to be an important dimension for risk stratification of patients with AKI after cardiac surgery [13]. As classification based on creatinine trajectories early in AKI course accounts for both absolute rise in creatinine and patient’s response to treatment, it could be a better tool for risk stratification of AKI. The role of classification of AKI based on early creatinine trajectories is unclear. Additionally, it is unknown whether such classification offers any advantages over current KDIGO AKI severity staging alone. We hypothesized that classification of AKI in critically ill patients with sepsis based on these trajectories will identify patients at risk for complications beyond KDIGO AKI staging.

## Methods

### Data sources

We used data from two independent databases—Medical Information Mart for Intensive Care IV (MIMIC-IV) and eICU Collaborative Research Database (eICU) [14, 15], for this study. MIMIC-IV is a single center database containing de-identified electronic health records of patients admitted to the Beth Israel Deaconess Medical Center from 2008 to 2019. We used the MIMIC-IV database, focusing on critically ill patients with sepsis who developed AKI within 48 h after their first ICU admission. Our aim was to develop AKI classification in critically ill patients with sepsis based on creatinine trajectories within the first 96 h (4 days) of ICU admission. We chose this time-period to adequately capture the early trajectory of the evolution of AKI. We further used this development cohort to develop prediction models to assess whether creatinine trajectories independently predict outcomes in sepsis patients with AKI. eICU is a multicenter database comprising de-identified health data from more than 200,000 ICU admissions across the United States during the period of 2014–2015. We used eICU as an independent external validation cohort.

### Study population

**Fig. 1:**
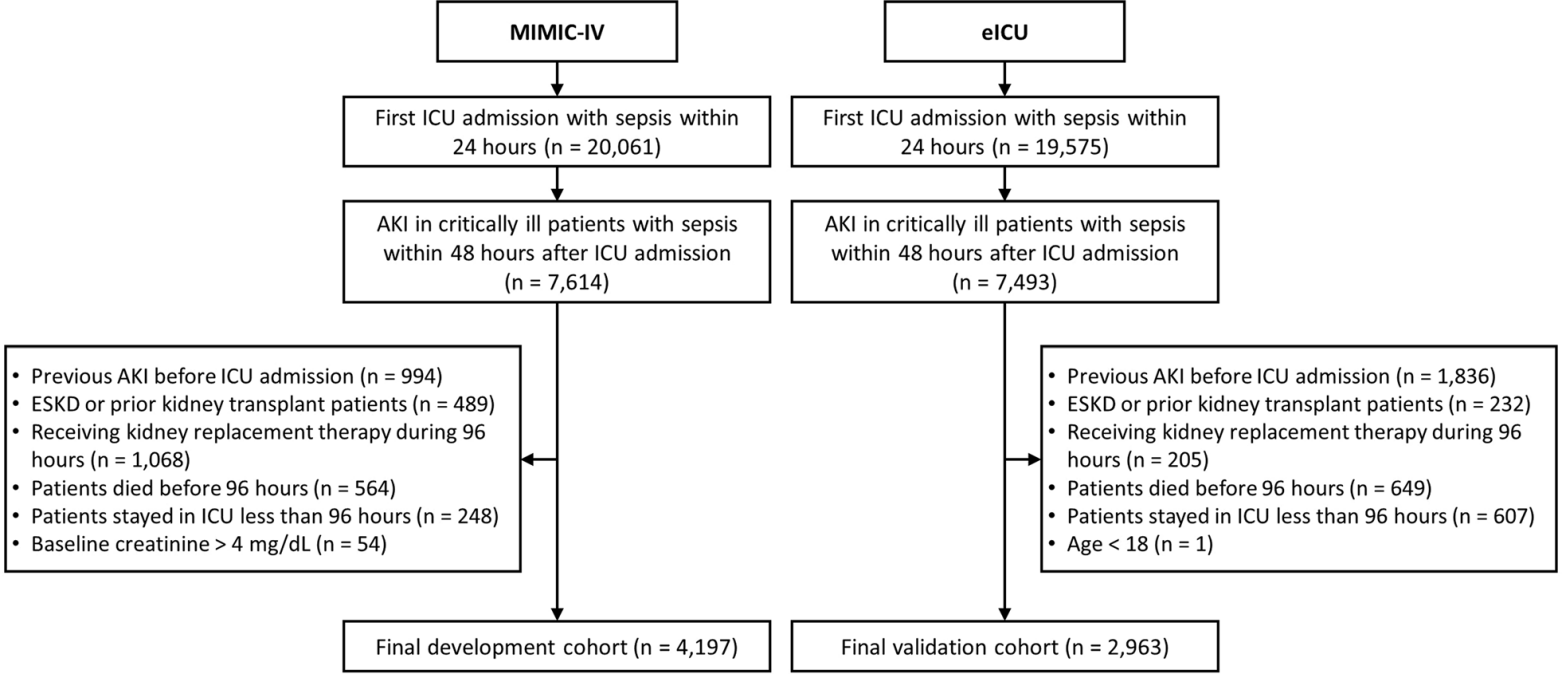
Consort diagram of development and validation cohorts

We included adult patients with sepsis who developed AKI within 48 h of intensive care unit (ICU) admission. We identified patients with sepsis based on a combination of suspicion of infection and increase in Sequential Organ Failure Assessment (SOFA) score by two or more within a 24-h period. To be consistent with prior literature [16, 17], we assumed a SOFA score of zero before ICU admission. We defined suspicion of infection as the co-occurrence of intravenous antibiotic treatment and collection of blood cultures such that if intravenous antibiotics were given first, then the cultures must have been obtained within 24 h [18]. In comparison, if cultures were obtained first, then intravenous antibiotics must have been ordered within 72 h. We identified onset of sepsis as the earlier of the suspicion of infection time and SOFA time if SOFA time occurred no more than 24 h before or 12 h after the suspected infection time [19]. As the eICU dataset provides limited data on body fluid samplings, we employed an alternative definition of suspected infection, which was determined by the occurrence of multiple antibiotic administrations. This alternative definition has been previously validated in a study comparing it to the original definition used in MIMIC-IV [20]. As per KDIGO guidelines, we defined AKI as an increase in serum creatinine by 0.3 mg/dL or more within 48 h or an increase by at least 1.5 times the reference serum creatinine within 7 days [8]. In congruence with previous literature [21] our specific inclusion criteria were—(1) Adult patients defined as those 18 years or older on admission, (2) who developed sepsis within 24 h of admission to ICU, and (3) developed AKI within 48 h after admission to ICU. For patients with multiple ICU admissions, we included data from only the first ICU admission. Figure S1 demonstrates the criteria to identify the definition and time of AKI in patients with sepsis onset [18–20]. We excluded patients under the age of 18 years, those with ESKD or prior kidney transplant, with a known baseline creatinine level > 4 mg/dL or with development of AKI prior to ICU admission. We also excluded patients receiving kidney replacement therapy or those who were discharged or died before 96 h after ICU admission. Details regarding the selection process employed in this study are given in Fig. 1.

### Outcome

The primary outcome was development of AKD, defined as the continued meeting of AKI criteria in surviving patients beyond 7 days after development of AKI [22]. The secondary outcomes were—(1) a composite of AKD or all-cause in-hospital mortality by day 7 after AKI onset, and (2) a composite of AKD by hospital discharge or all-cause in-hospital mortality.

### Feature extraction

We included data on patient demographics, comorbidities, baseline creatinine, laboratory values, vasopressor use and duration and exposure to nephrotoxic drugs [23] during the first 96 h of the ICU admission. Similar to prior literature, we determined baseline serum creatinine as the median serum creatinine within 12 months prior to hospital admission [24, 25]. For patients with missing previous serum creatinine values in this timeframe, as recommended by KDIGO, we estimated the baseline serum creatinine by assuming the Modification of Diet in Renal Disease estimated glomerular filtration rate of 75 mL/min per 1.73 m^2^ [8]. In accordance with previous studies, we then determined the reference serum creatinine as the lower of the baseline serum creatinine mentioned above and the first admission serum creatinine [24, 26]. Following published literature, we excluded variables with more than 40% missing values [27–29]. All other missing data were imputed using predictive mean matching techniques with five imputations based on Multivariate Imputation via Chained Equations (MICE) function in R [30].

### Statistical analysis

We expressed descriptive results for the participant baseline characteristics as either mean with standard deviation or as median with interquartile range, depending on skewness. We compared categorical features using Chi-square test and continuous features using Student t test and Mann–Whitney U tests.

We utilized the latent class mixed models (LCMM) to identify classes based on serum creatinine trajectories defined by the percentage change in serum creatinine as ([serum creatinine–reference creatinine]/reference creatinine)*100. LCMM is a robust and validated statistical method designed to uncover clinically significant patient subgroups with similar longitudinal outcomes [31]. It integrates mixed models, which track individual-level growth in longitudinal outcomes through random effects, with latent class analysis that classifies patients into groups based on shared progression patterns. We calculated creatinine changes. We developed LCMM models with varying number of classes (2–10) in the development cohort. We selected the model with the lowest Bayesian Information Criterion (BIC). Subsequently, we computed the probability that a patient belongs to each class using this model and classified them into the class to which they had the highest probability of belonging. We assessed model discrimination using the mean posterior class membership probability (MPCMP). It is a class-specific metric that represents the mean probability of class membership for patients assigned to that class [32]. We then used mixed models to estimate marginal projection of the changes in creatinine levels across different LCMM classes. For external validation, we applied the trained LCMM model to the validation cohort, utilizing all estimated parameters derived from the model trained on the development cohort to classify creatinine trajectories with the validation cohort.

We used regression models to identify the impact of the classification of AKI in critically ill patients with sepsis based on early creatinine trajectories on various outcomes. We used logistic regression to assess the relationship between classification of AKI in critically ill patients with sepsis based on early creatinine trajectories and development of AKD. We used Kaplan–Meier and Cox regression analyses to evaluate the relationship between classification of AKI in critically ill patients with sepsis based on early creatinine trajectories and composite outcomes of AKD or all-cause in-hospital mortality by day 7, and AKD or all-cause in-hospital mortality by hospital discharge. To evaluate the independent effect of classification of AKI in critically ill patients with sepsis based on early creatinine trajectories on these outcomes, we adjusted the logistic and Cox regressions for age, gender, race, comorbidities, laboratory results, baseline creatinine level, SOFA score, initial and maximum AKI staging. We performed all analyses using R, version 4.2.2 [33].

## Results

A total of 4197 patients with AKI in critically ill patients with sepsis from MIMIC-IV database satisfied the inclusion and exclusion criteria of this study and served as the development set. A total of 3963 patients with AKI in critically ill patients with sepsis from eICU served as external validation set. A comparison of baseline characteristics and outcomes between two cohorts is shown in Tables 1 and 2 respectively.

**Table 1:** Baseline characteristics

	MIMIC-IV (4197)	eICU (3963)	*p*-value
Age (years)	70 (59, 80)	67 (59, 78)	0.001
Male (%)	2577 (61%)	2228 (56%)	< 0.001
Race (%)			< 0.001
White	2883 (69%)	3171 (80%)	
Black	375 (9%)	434 (11%)	
Hispanic	139 (3%)	64 (2%)	
Others	800 (19%)	294 (7%)	
Height (cm)	170 (163, 178)	170 (163, 178)	0.07
Weight (kg)	80 (68, 96)	84.7 (70, 104)	< 0.001
Underlying diseases (%)			
Diabetes mellitus	951 (23%)	467 (12%)	< 0.001
Congestive heart failure	968 (23%)	560 (14%)	< 0.001
Arrhythmia	774 (18%)	587 (15%)	< 0.001
Chronic lung disease	620 (15%)	379 (10%)	< 0.001
Liver disease	328 (8%)	180 (5%)	< 0.001
Laboratory^a^			
Hemoglobin (g/dL)	8.4 (7.5, 9.6)	8.8 (7.6, 10.2)	< 0.001
Hematocrit (%)	25 (23, 29)	27 (23, 31)	< 0.001
White blood cell count (× 10^9^/L)	15.4 (11.5, 20.5)	15.3 (11.3, 20.8)	0.54
Platelet (× 1000/mm^3^)	123 (82, 180)	130 (85, 185)	0.02
PT	16 (14, 20)	17 (14, 22)	< 0.001
PTT	37 (31, 55)	38 (32, 55)	0.22
INR	1.5 (1.3, 1.9)	1.4 (1.2, 2)	0.001
BUN (mg/dL)	38 (26, 57)	44 (31, 61)	< 0.001
Sodium (mmo/L)	142 (139, 145)	142 (139, 146)	< 0.001
Potassium (mmo/L)	4.8 (4.4, 5.2)	4.7 (4.3, 5.1)	< 0.001
Chloride (mmo/L)	109 (106, 113)	109 (105, 114)	0.97
Bicarbonate (mmo/L)	20 (17, 23)	20 (16, 23)	0.01
Calcium (mg/dL)	8.6 (8.2, 9)	8.6 (8.1, 9)	< 0.001
SGOT (U/L)	54 (28, 137)	46 (25, 118)	< 0.001
SGPT (U/L)	34 (18, 100)	31 (18, 79)	0.01
ALP (U/L)	86 (62, 129)	84 (61, 125)	0.21
Albumin (d/dL)	2.9 (2.4, 3.2)	2.5 (2.1, 2.9)	< 0.001
Glucose (mg/dL)	171 (140, 229)	183 (147, 246)	< 0.001
pH	7.30 (7.24, 7.35)	7.31 (7.24, 7.37)	< 0.001
pO2	64 (40, 87)	75 (62, 95)	< 0.001
pCO2	47 (42, 55)	43 (37, 52)	< 0.001
pO2/FiO2	319 (234, 414)	310 (217, 376)	< 0.001
Lactate (mg/dL)	2.4 (1.7, 3.7)	2 (1.3, 3.6)	< 0.001
Baseline creatinine (mg/dL)^b^	1 (0.8, 1.2)	0.98 (0.8, 1)	< 0.001
First AKI staging (%)			< 0.001
1	2755 (66%)	2047 (52%)	
2	667 (16%)	1114 (28%)	
3	775 (18%)	802 (20%)	
MAX AKI Staging (%)			< 0.001
1	2421 (58%)	1508 (38%)	
2	788 (19%)	1204 (30%)	
3	988 (23%)	1251 (32%)	
MAX SOFA score	7 (5, 10)	8 (5, 10)	< 0.001
Vasopressor use (%)^c^	2603 (62%)	1019 (26%)	< 0.001
Vasopressor duration (minutes)	1285 (419, 3101)	2731 (960, 4906)	< 0.001
Nephrotoxins (%)	1441 (34%)	2883 (73%)	< 0.001

Data are presented as count (percent) or median (interquartile range [IQR])

*AKI* acute kidney injury, *BUN* blood urea nitrogen, *SGPT* serum glutamic pyruvic transaminase, *SGOT* serum glutamic-oxaloacetic transaminase, *SOFA* sequential organ failure assessment

^a^Laboratory values were selected by the most abnormal results

^b^Baseline serum creatinine was determined as the lower of the median serum creatinine level within 12 months prior to hospital admission and the first admission creatinine measure. For patients with missing previous serum creatinine level, we calculated a baseline serum creatinine level by using the Modification of Diet in Renal Disease equation as recommended in the KDIGO AKI guideline, assuming a glomerular filtration rate of 75 mL/min per 1.73 m^2^

^c^Vasopressors included norepinephrine, dopamine, epinephrine, phenylephrine, or vasopressin

**Table 2:** Clinical outcomes

	MIMIC-IV (4197)	eICU (3963)	*p*-value
Outcomes			
AKD day 7 (%)	674 (16%)	1000 (25%)	< 0.001
Death within 7 days (%)	198 (5%)	184 (5%)	0.87
In hospital mortality (%)	591 (14%)	512 (13%)	0.12
Composite of AKD and death by day 7 (%)	872 (21%)	1184 (30%)	< 0.001
Composite of AKD or in-hospital mortality by hospital discharge (%)	772 (18%)	1008 (25%)	< 0.001
ICU length of stay (days)	3.9 (2, 7.2)	4.1 (2.2, 7.4)	0.004
Hospital length of stay after ICU admission (days)	9.2 (6.1, 15.4)	9.1 (6.3, 14.4)	0.002

### Classification of AKI in critically ill patients with sepsis based on early creatinine trajectories

**Fig. 2:**
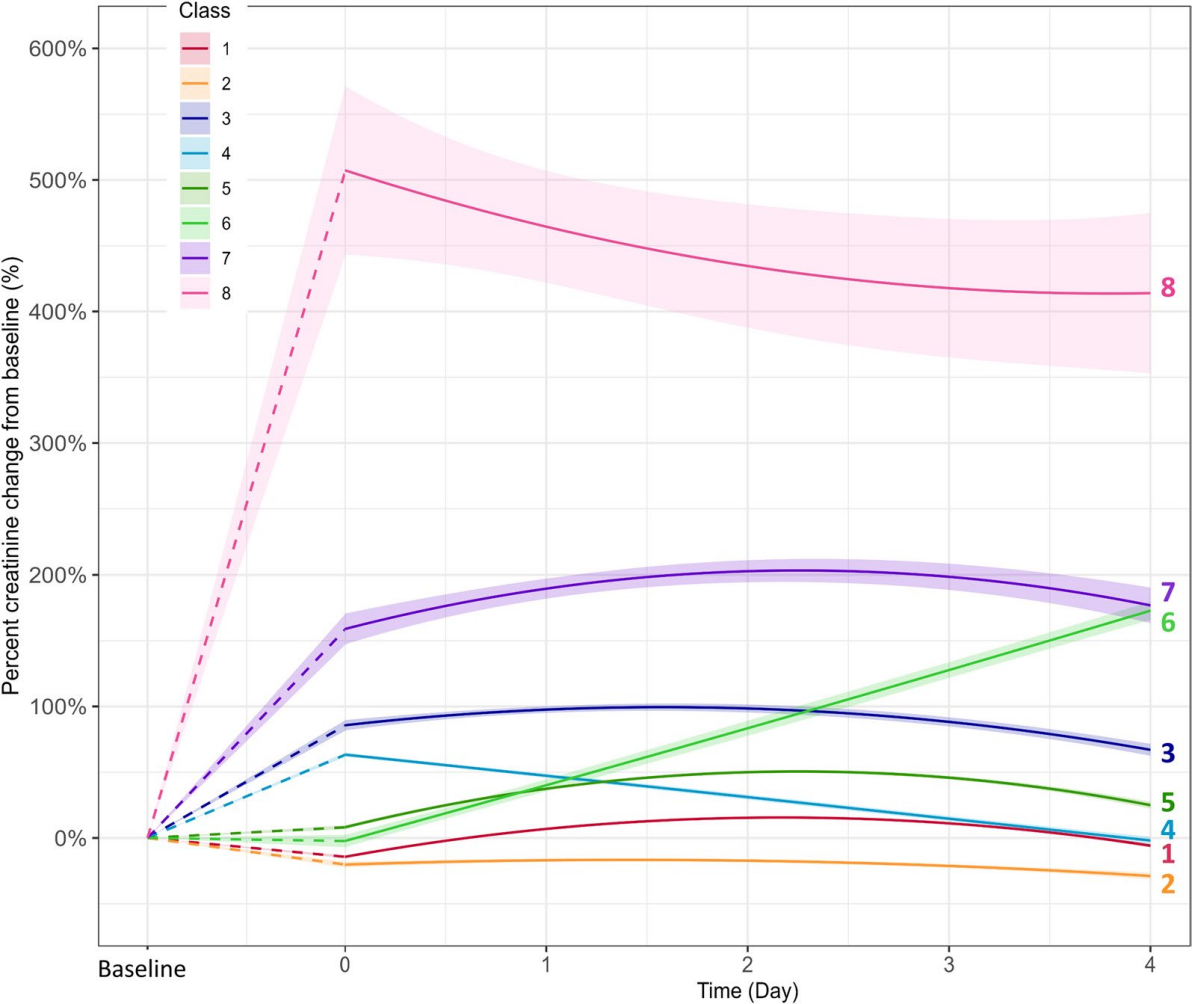
AKI in critically ill patients with sepsis classification based on serum creatinine trajectories in development cohort

With the lowest BIC for an eight-class model (Additional file 2: Table S1), we identified 8 distinct Classes of creatinine trajectories in the development cohort using LCMM. MPCMP values for class assignment ranged from 62 to 89% (Additional file 2: Table S2), indicating good discrimination between classes. These classes showed differences in AKI staging, rate, timing, and recovery (Fig. 2).

- *Class 1. Transient AKI*—Creatinine trajectory in this class showed a minor nadir from baseline on ICU admission, followed by a mild rise that peaked at AKI stage 1 on day 2–3, with subsequent recovery.
- *Class 2. Minor Transient AKI*—Creatinine trajectory in this class started with a minor decrease from baseline, followed by a mild rise in creatinine that peaked on day 1, with subsequent recovery of AKI.
- *Class 3. Early Mild AKI with Persistence*—Creatinine trajectory in this class started with stage I AKI at ICU admission with continued rise in creatinine till day 1 to 2, followed by a sustained decline but persistence of AKI.
- *Class 4. Early Mild AKI followed by Recovery*—Creatinine trajectory in this class started with an initial stage 1 AKI on admission to ICU which then showed a sustained decline to AKI resolution.
- *Class 5. Late Mild AKI with persistence*—This class had a mild rise in creatinine on ICU admission that continued worsening with peak at AKI stage 1 by day 2, followed by a sustained decline but persistence of AKI.
- *Class 6. Late Mild AKI with persistence and worsening*—This class had a mildly elevated creatinine on ICU admission that then showed a sustained increase.
- *Class 7. Moderate AKI with persistence*—This class had stage II AKI on ICU admission with continued increased in creatinine for a peak of AKI stage 2–3 by day 2–3. This was followed by a mild decline but with persistence of AKI.
- *Class 8. Severe AKI with mild improvement but persistence*—This class had AKI stage 3 on ICU admission followed by a small, sustained decline thereafter but with continued persistence of AKI.

The class observed most was Transient AKI (Class 1) (23.5%), followed by Late Mild AKI with Persistence (Class 5) (22%) and Early Mild AKI with Persistence (Class 3) (17%). There were significant differences among Classes in age (*p* < 0.001), hemoglobin (*p* = 0.009), white blood cell counts (*p* = 0.02), lactate levels (*p* = 0.009), maximum SOFA score (*p* < 0.001), vasopressor duration (*p* < 0.001) (Additional file 2: Table S3).

### Classification of AKI in critically ill patients with sepsis based on early creatinine trajectories and outcomes in the development cohort (MIMIC-IV)

#### Development of AKD

**Fig. 3:**
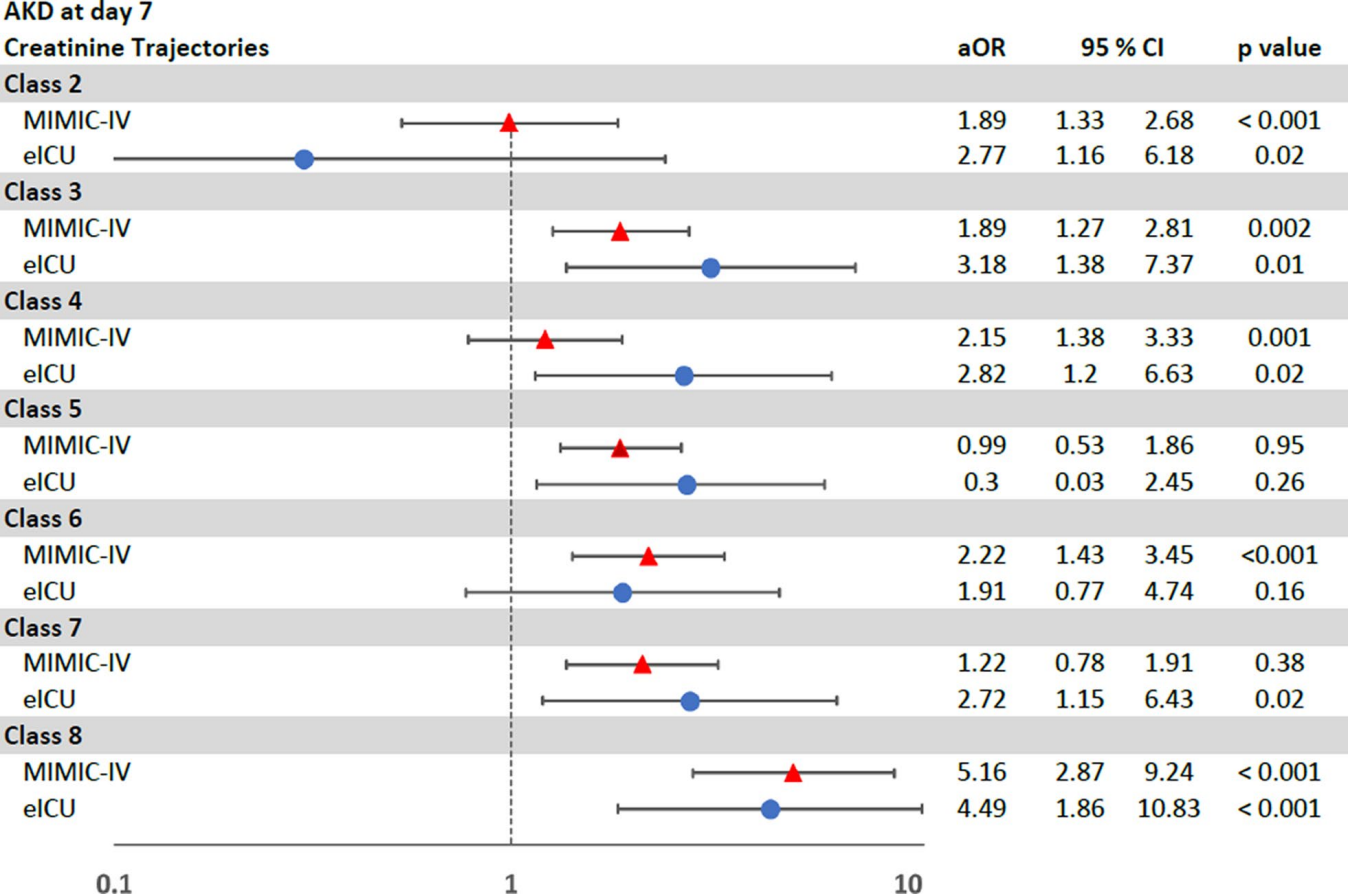
Multivariable logistic regression analysis of the risk of AKD

In development cohort, 674 (16%) patients developed AKD (Table 2). There were significant differences among Classes in development of AKD (*p* < 0.001) (Additional file 2: Table S4). On regression analysis, the creatinine trajectories were associated with differing risks of development of AKD. In comparison to patients with transient AKI (Class 1), the highest risk for development of AKD was seen in patients in severe AKI with mild improvement but persistence (Class 8) (OR 9.02; 95% CI 5.94–13.7; *p* < 0.001), followed by Class 6, 7, 3, and 5 respectively (Table 3). This difference in the risks of development of AKD by classes based on creatinine trajectories persisted on multivariable logistic regression analysis where Class 8 still showed the highest risk for development of AKD with Class 1 as the reference group (OR 5.16; 95% CI 2.87–9.24; *p* < 0.001) (Fig. 3).

**Table 3:** Univariate logistic regression models for AKD and univariate cox regression models for composite outcomes in the development cohort

	AKD			AKD or mortality by day 7			AKD at discharge or in-hospital mortality		
	OR	95% CI	*p*	HR	95% CI	*p*	HR	95% CI	*p*
Class 1 (reference group)	1			1			1		
Class 2	0.99	0.60, 1.63	0.97	0.99	0.68, 1.47	0.99	1.14	0.82, 1.58	0.43
Class 3	3.99	2.87, 5.55	< 0.001	2.31	1.75, 3.04	< 0.001	1.51	1.17, 1.94	0.001
Class 4	1.30	0.85, 2.01	0.23	0.85	0.58, 1.23	0.38	0.57	0.39, 0.83	0.003
Class 5	2.52	1.80, 3.55	< 0.001	1.92	1.45, 2.54	< 0.001	1.32	1.02, 1.70	0.03
Class 6	7.11	4.81, 10.53	< 0.001	4.98	3.60, 6.89	< 0.001	3.48	2.56, 4.74	< 0.001
Class 7	5.59	3.99, 7.83	< 0.001	3.02	2.28, 3.99	< 0.001	1.79	1.37, 2.33	< 0.001
Class 8	9.02	5.94, 13.69	< 0.001	3.38	2.36, 4.85	< 0.001	1.93	1.31, 2.82	< 0.001

#### Composite of AKD or mortality in seven days

In the development cohort, 872 (21%) patients developed AKD or died within seven days after AKI onset.

On univariable cox regression analysis, creatinine trajectories were associated with differing risks of development of a composite of AKD or all-cause in-hospital mortality by day 7 after AKI onset. In comparison to Transient AKI (Class 1), Late Mild AKI with Persistence and Worsening (Class 6) showed the highest risk for development of a composite of AKD or mortality in 7 days (HR 4.98; 95% CI 3.60–6.89; *p* < 0.001), followed by Severe AKI with Mild Improvement but Persistence (Class 8) (HR 3.38; 95% CI 2.36–4.85; *p* < 0.001) and Moderate AKI with Persistence (Class 7) (HR 3.02; 95% CI 2.28–3.99; *p* =  < 0.001), respectively (Table 3 and Additional file 1: Figure S2).

**Fig. 4:**
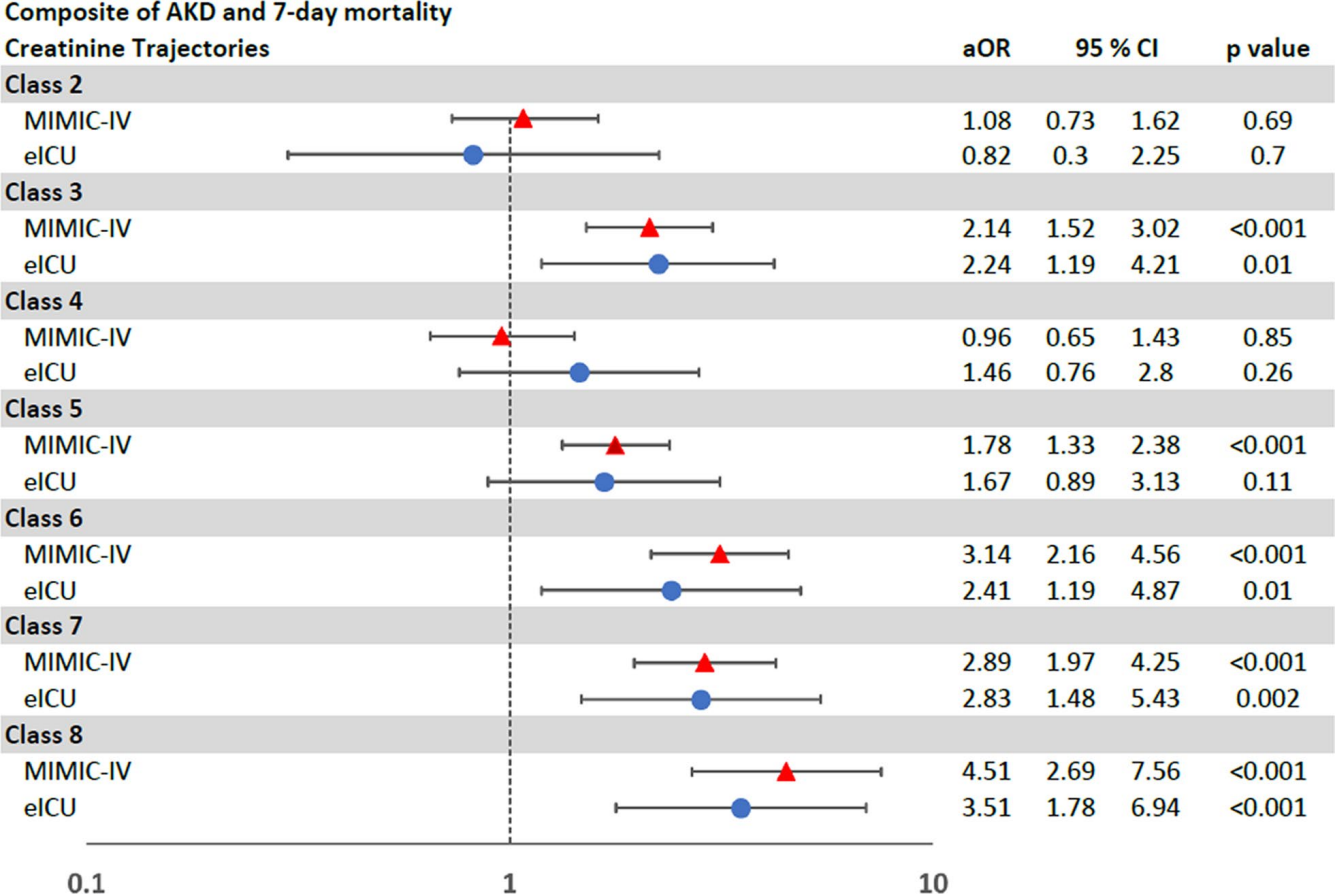
Multivariable Cox regression analysis of a composite of AKD and 7-day mortality

This difference in the risks of development of a composite of AKD or all-cause in-hospital mortality by day 7 after AKI onset by classes based on creatinine trajectories persisted on adjusted survival analysis (Fig. 4).

#### Composite of AKD or total all-cause in-hospital mortality by discharge

**Fig. 5:**
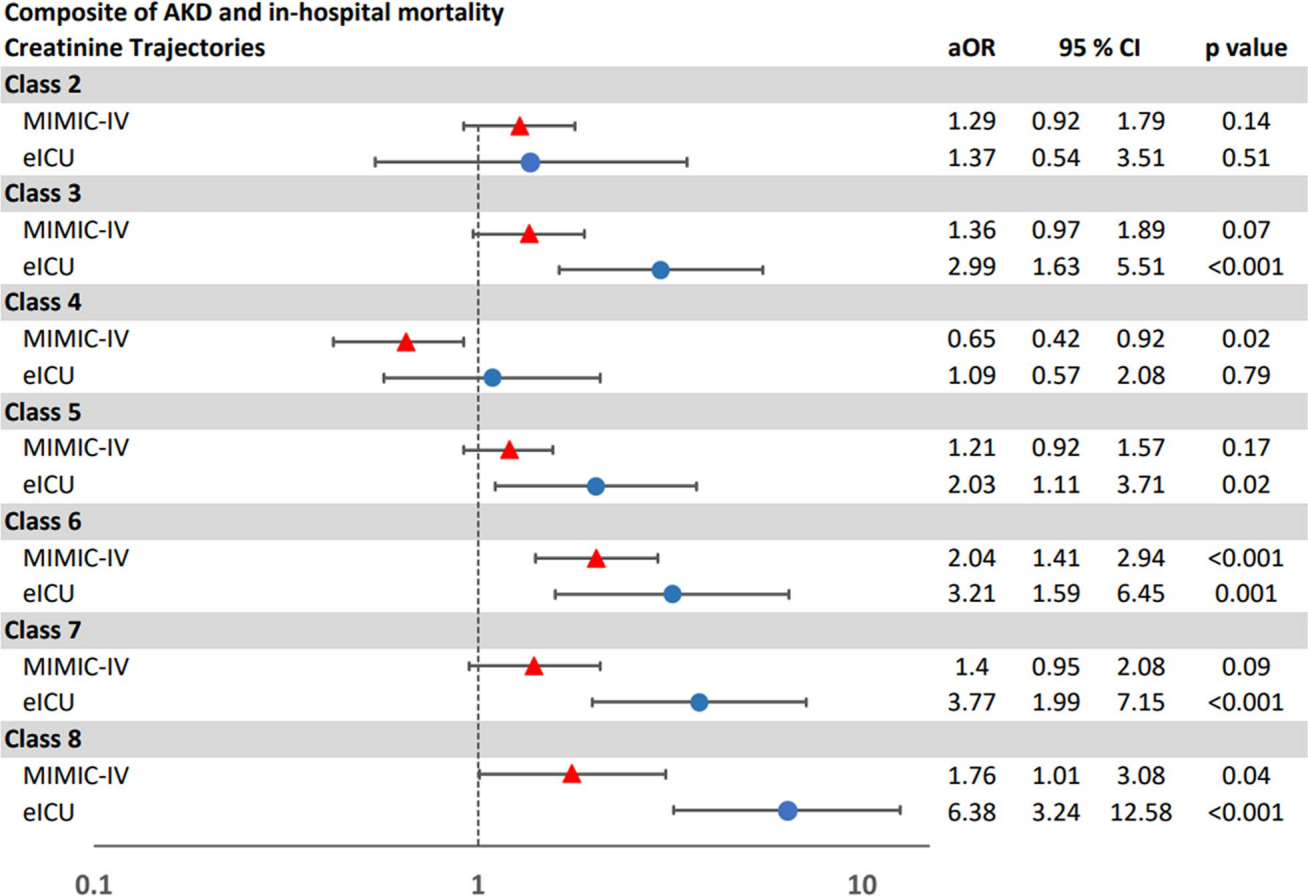
Multivariable Cox regression analysis of a composite of AKD at discharge and in-hospital mortality

A total 772 (18%) patients had AKD by discharge or died during the hospital admission in the development cohort (Table 2). The classes of creatinine trajectories were associated with differing risks of development of a composite of AKD or all-cause in-hospital mortality by discharge in both unadjusted (Table 3 and Additional file 1: Figure S3) and adjusted analyses (Fig. 5).

### Validation of creatinine trajectories in independent external cohort (eICU)

We validated the LCMM model and identified 8 creatinine trajectories in the eICU which served as our external validation cohort. Discrimination among classes was good, with MPCMP ranging from 60 to 91% (Additional file 2: Table S5). There were significant differences among Classes in age (*p* < 0.001), hemoglobin (*p* < 0.001), white blood cell counts (*p* < 0.001), lactate levels (*p* < 0.001), maximum SOFA score (*p* < 0.001), vasopressor duration (*p* < 0.001), and development of AKD (*p* < 0.001) (Additional file 2: Table S6, S7).

Among 3963 AKI with sepsis patients, 1000 (25%) patients developed AKD at day 7 (Table 2). Like the development cohort, these classes of creatinine trajectories in the external validation cohort were associated with differing risks of development of AKD, and composite of both AKD or all-cause in-hospital morality by day 7 and AKD or all-cause in-hospital by discharge, in both unadjusted and adjusted analyses (Table 4 and Figs. 3, 4, 5).

**Table 4:** Univariate logistic regression models for AKD and univariate cox regression models for composite outcomes in the validation cohort

	AKD			AKD or mortality by day 7			AKD at discharge or in-hospital mortality		
	OR	95% CI	*p*	HR	95% CI	*p*	HR	95% CI	*p*
Class 1 (reference group)	1			1			1		
Class 2	0.39	0.05, 3.21	0.38	0.90	0.33, 2.44	0.84	1.24	0.49, 3.16	0.65
Class 3	7.58	3.38, 17.01	< 0.001	3.57	1.96, 6.49	< 0.001	3.46	1.95, 6.16	< 0.001
Class 4	2.53	1.07, 5.95	0.03	1.47	0.77, 2.82	0.24	1.16	0.61, 2.21	0.65
Class 5	3.69	1.61, 8.45	0.002	1.92	1.03, 3.58	0.04	2.25	1.24, 4.09	0.007
Class 6	9.26	3.85, 22.32	< 0.001	4.47	2.28, 8.76	< 0.001	4.59	2.37, 8.88	< 0.001
Class 7	13.32	5.95, 29.83	< 0.001	4.23	2.33, 7.69	< 0.001	3.61	2.03, 6.42	< 0.001
Class 8	19.97	8.79, 45.4	< 0.001	4.66	2.52, 8.62	< 0.001	4.82	2.66, 8.74	< 0.001

## Discussion

In this study using data from two large, independent critical care databases we derived and validated 8 different classes of early creatinine trajectories early in AKI in critically ill patients with sepsis. These classes were heterogenous in both baseline characteristics and outcomes of patients with AKI in critically ill patients with sepsis. Additionally, we show that membership in these classes is an independent predictor for AKD and composite of AKD or mortality by day 7 and composite of AKD or mortality by hospital discharge.

AKI is common in critically ill patients and is associated with high morbidity and mortality. However, not all instances of AKI are the same as is demonstrated by the recognition of various stages of AKI [8]. The current staging for AKI relies on the severity of AKI as identified by maximum change in serum creatinine or minimum urine output over a period. The recent discovery of different phenotypes of AKI [9] suggests that classification of AKI just by severity is inadequate, particularly when considering different etiologies of AKI and effects of early management strategies of AKI in patients with sepsis [9, 25]. Use of an unbiased methodology to identify novel and clinically relevant sub-phenotypes of AKI based on early trajectory of serum creatinine provides a novel approach to classify AKI in critically ill patients with sepsis. An added advantage of this technique is that it enhances and further personalizes the current AKI classification, which relies solely on its severity.

The trajectories of serum creatinine have been shown to be associated with different outcomes in patients with AKI. Bhatraju et al. [34] studied the creatinine trajectory in first 72 h of ICU stay among critically ill patients with AKI and showed that patients who have a non-resolving AKI subphenotype in that timeframe have a higher risk of mortality in comparison to patients with a resolving subphenotype of AKI (RR 1.68; 95% CI 1.15–2.33). Similarly, Kellum et al. [35] delineated five distinct recovery sub-phenotypes among critical care patients with AKI during the first week following the onset of AKI. These sub-phenotypes were classified based on patterns of reversal, relapse, or recovery. Sub-phenotypes with patients who never recovered or had reversal with relapse had the longest lengths of stay and the worst prognosis, while patients recovering late did better than those with no recovery, but not as well as those recovering early.

Most recently, Andrew et al. [13] employed LCMM technique to identify twelve different trajectories of serum creatinine within the first 4 days after cardiac surgery. Among them, they found that there were four creatinine trajectories that were associated with higher risk of death. These results are similar to our study where we identified eight creatinine trajectories, with distinct clinical characteristics and outcomes, in patients with AKI in critically ill patients with sepsis. With increasing recognition of the importance of AKD, we focused our attention in this study on studying the impact of these trajectories on development of AKD.

Development of AKD is associated with development of CKD, ESKD, longer length of stay, and greater risk of mortality [36, 37]. Additionally, the AKD period itself represents a critical time window during which interventions could be initiated to potentially alter the natural history of kidney disease. Though mortality is a critical endpoint, use of AKD for risk stratification of AKI in critically ill patients allows for a more holistic assessment by incorporating both risk for increased mortality with AKD and increased risk for further decline in kidney function. Additionally, it allows for identification of high-risk patients where targeted novel therapies could decrease the onset of AKD, and subsequent long term adverse consequences. In this study, we show that classification based on early creatinine trajectories in sepsis patients with AKI identifies patients at risk for AKD. Moreover, these classes further identify patients at risk for a composite of AKD or mortality by day 7 and AKD or mortality by discharge. Risk stratification of patients with AKI in critically ill patients with sepsis by creatinine trajectories is independent of AKI staging, thus providing a method to further personalize care. It has been shown that certain patients with AKI respond differently to vasopressin therapy [38]. As classification by creatinine trajectories incorporates information about both severity of AKI and its response to early management strategies, this may allow for further identification of differential responses to therapeutic interventions across these classes. This classification would also be important for timely allocation of appropriate resources for patients, such as need for follow up in nephrology clinic on discharge.

It is important to acknowledge the limitations of this study. Most importantly, this is a retrospective study and the impact of these classes on clinical care needs to be evaluated in prospective studies. Furthermore, our study aims to classify early AKI in critically ill patients with sepsis based on initial 96-h serum creatinine trajectory after ICU admission. We, therefore, utilized creatinine-based criteria to define AKI, while focusing on patients who developed AKI within first 48 h of ICU admission and survived the initial 96 h. As kidney replacement therapy directly affects serum creatinine measurements, we excluded patients receiving kidney replacement therapy during the first 96 h after ICU admission. Despite these limitations, our study is a significant step towards personalization of risk stratification of AKI in critically ill patients with sepsis.

## Conclusions

We identified eight distinct classes of AKI in critically ill patients with sepsis based-on serum creatinine trajectories within the first 96 h after AKI. These classes identified patients with distinct clinical characteristics and risk for development of AKD, AKD or mortality by day 7 and AKD or mortality by hospital discharge. This risk stratification by creatinine trajectories was independent of AKI staging and validated independently. Further studies are needed to identify therapeutic and management implications of creatinine trajectories.

### Supplementary Information

Supplementary Material 1: Figure S1. Criteria to identify time of AKI in critical ill patients with sepsis onset. Figure S2. Kaplan Meier survival curve for AKD or 7-day mortality for creatinine trajectories in development cohort (A) and validation cohort (B). Figure S3. Kaplan Meier survival curve for AKD at discharge or in-hospital mortality in development cohort (A) and validation cohort (B).

Supplementary Material 2: Table S1. Fit statistics for latent class mixed models in development cohort. Table S2. Mean Posterior Probability of Membership in Class in MIMIC-IV. Table S3. Baseline characteristics by creatinine trajectory in MIMIC-IV. Table S4. Clinical outcomes by creatinine trajectory in MIMIC-IV. Table S5. Mean Posterior Probability of Membership in Class in eICU database. Table S6. Baseline characteristics by creatinine trajectory in eICU database. Table S7. Clinical outcomes by creatinine trajectory in eICU database.

## Data Availability

Publicly available datasets were analyzed in this study. The dataset used in this study, MIMIC-IV, is available at https://mimic.physionet.org/, and the eICU dataset is available at https://github.com/mit-lcp/eicu-code.

## Abbreviations

AKD: Acute kidney disease
AKI: Acute kidney injury
BIC: Bayesian information criterion
CKD: Chronic kidney disease
eICU: Electronic Intensive Care Unit
ESKD: End-stage kidney disease
ICU: Intensive care unit
KDIGO: Kidney Disease Improving Global Outcomes
LCMM: Latent class mixed models
MICE: Multivariate imputation via chained equations
MIMIC-IV: Medical Information mart for intensive care IV
MPCMP: Mean posterior class membership probability
