# Supplementary material for: Machine learning derived serum creatinine trajectories in acute kidney injury in critically ill patients with sepsis
Source: Crit Care. 2024 May 10;28:156. doi: 10.1186/s13054-024-04935-x (PMC11084026; doi:10.1186/s13054-024-04935-x)
Supplement: Supplementary file 1 — Supplementary Material 1: Figure S1. Criteria to identify time of AKI in critical ill patients with sepsis onset. Figure S2. Kaplan Meier survival curve for AKD or 7-day mortality for creatinine trajectories in development cohort (A) and validation cohort (B). Figure S3. Kaplan Meier survival curve for AKD at discharge or in-hospital mortality in development cohort (A) and validation cohort (B). [file 13054_2024_4935_MOESM1_ESM.docx]

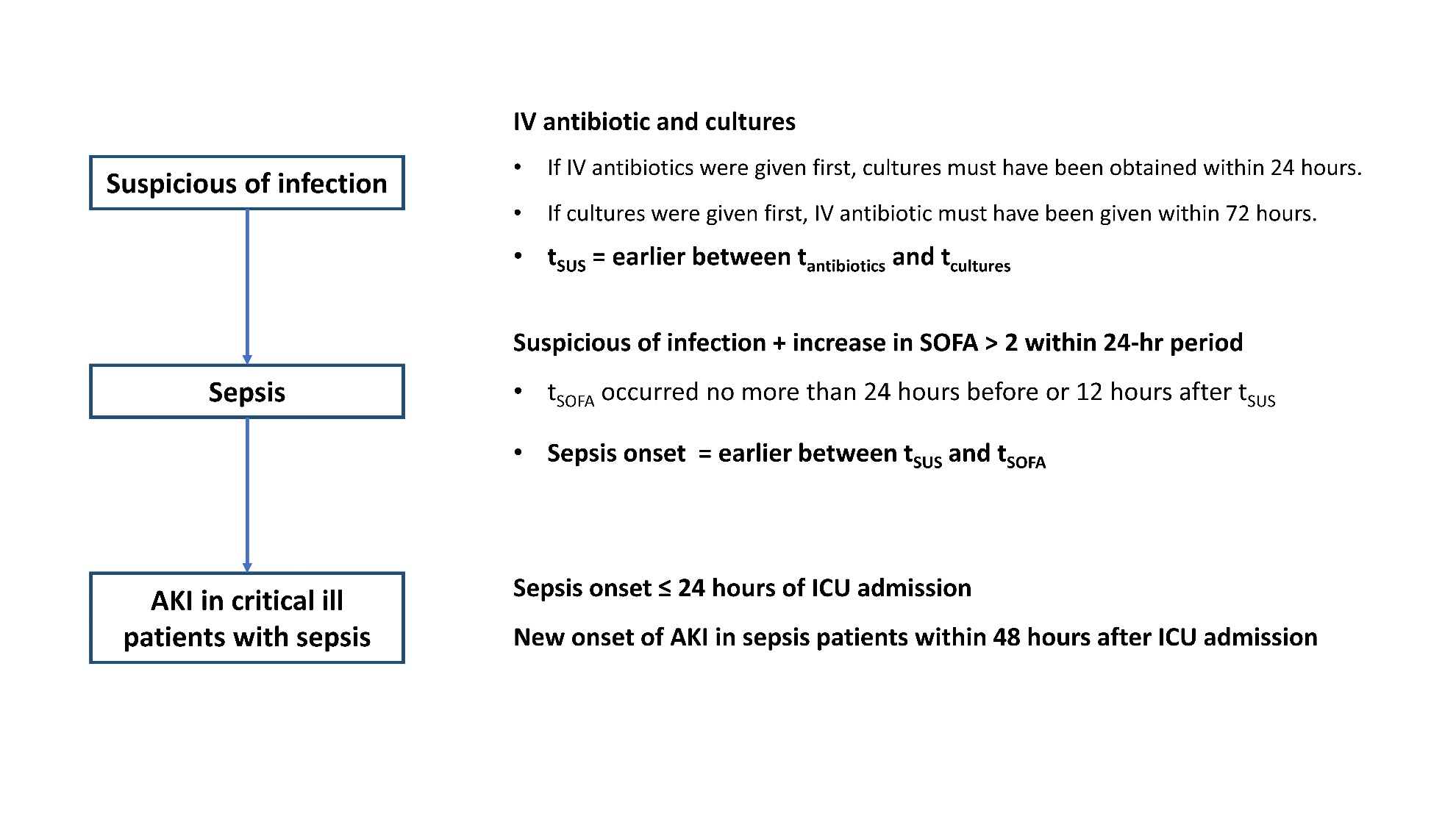


**Figure S1: Criteria to identify time of AKI in critical ill patients with sepsis onset**

**
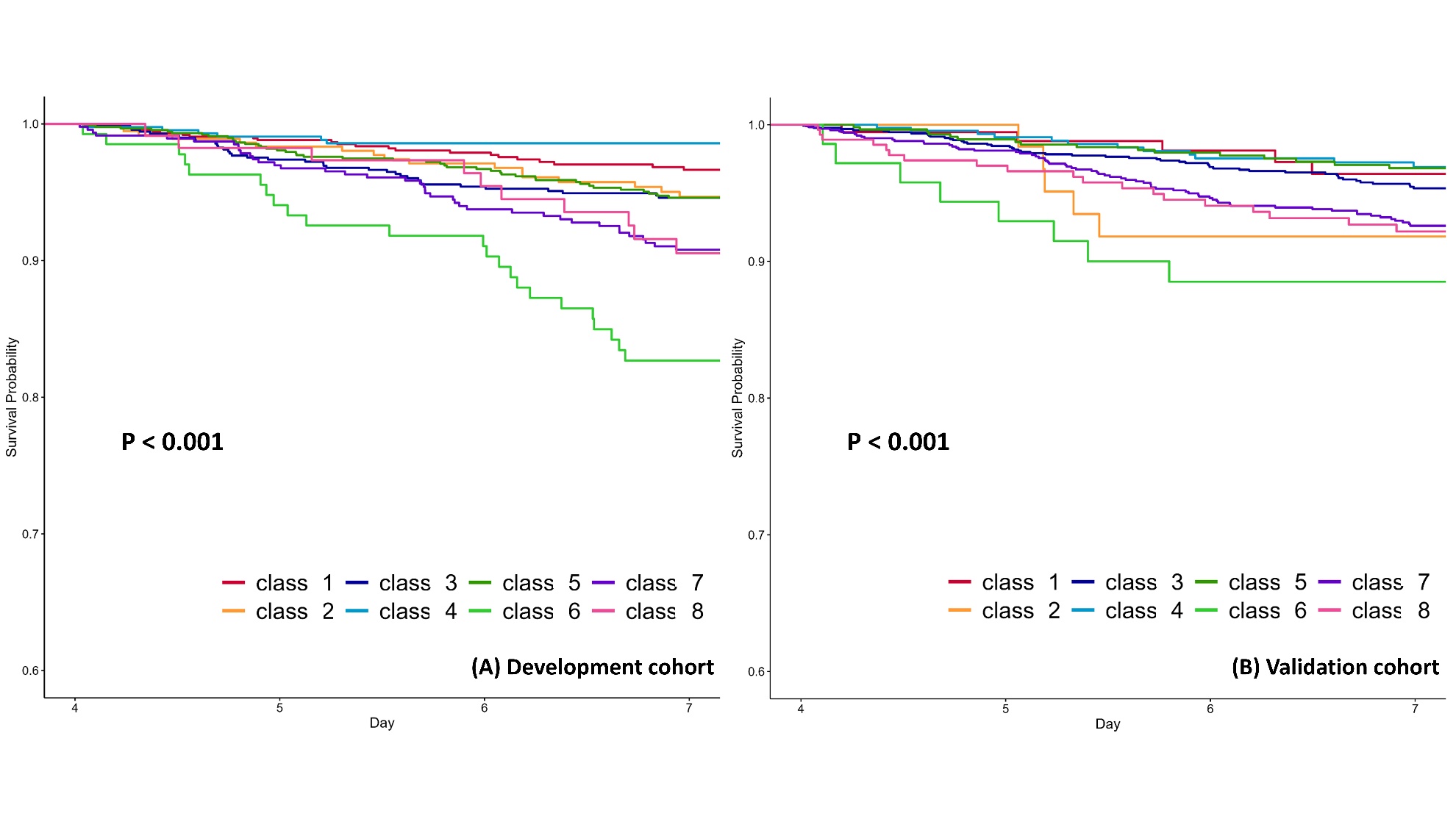
**

**Figure S2. Kaplain Meier survival curve for AKD or 7-day mortality for creatinine trajectories in development cohort (A) and validation cohort (B)**

**
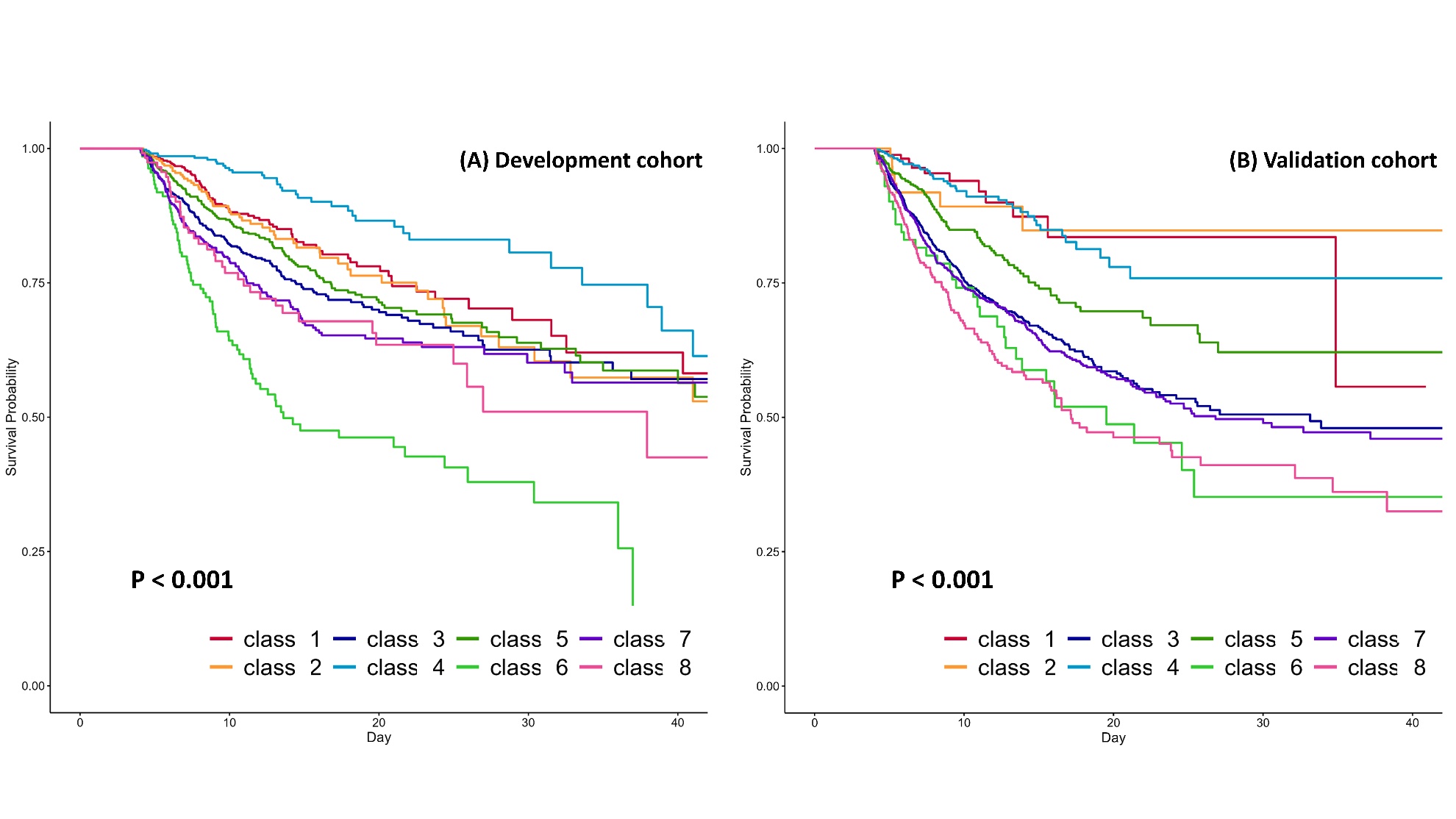
**

**Figure S3. Kaplain Meier survival curve for AKD at discharge or in-hospital mortality in development cohort (A) and validation cohort (B)**
