# Supplementary material for: Machine learning derived serum creatinine trajectories in acute kidney injury in critically ill patients with sepsis
Source: Crit Care. 2024 May 10;28:156. doi: 10.1186/s13054-024-04935-x (PMC11084026; doi:10.1186/s13054-024-04935-x)
Supplement: Supplementary file 2 — Supplementary Material 2: Table S1. Fit statistics for latent class mixed models in development cohort. Table S2. Mean Posterior Probability of Membership in Class in MIMIC-IV. Table S3. Baseline characteristics by creatinine trajectory in MIMIC-IV. Table S4. Clinical outcomes by creatinine trajectory in MIMIC-IV. Table S5. Mean Posterior Probability of Membership in Class in eICU database. Table S6. Baseline characteristics by creatinine trajectory in eICU database. Table S7. Clinical outcomes by creatinine trajectory in eICU database. [file 13054_2024_4935_MOESM2_ESM.docx]

**Table S1. Fit statistics for latent class mixed models in development cohort**

| **Number of classes** | **Bayesian information criteria** |
| --- | --- |
| 2 | 13633 |
| 4 | 11702 |
| 6 | 11438 |
| **8** | **11425** |
| 10 | 11430 |

**Table S2. Mean Posterior Probability of Membership in Class in MIMIC-IV**

| **Class** | **1** | **2** | **3** | **4** | **5** | **6** | **7** | **8** |
| --- | --- | --- | --- | --- | --- | --- | --- | --- |
| **1** | **0.67** | 0.13 | 0.01 | 0.02 | 0.17 | 0 | 0 | 0 |
| **2** | 0.16 | **0.76** | 0.02 | 0.01 | 0.05 | 0 | 0.01 | 0 |
| **3** | 0 | 0 | **0.72** | 0.05 | 0.06 | 0.04 | 0.13 | 0 |
| **4** | 0.01 | 0.01 | 0.18 | **0.62** | 0.17 | 0 | 0.01 | 0 |
| **5** | 0.09 | 0 | 0.13 | 0.1 | **0.66** | 0.01 | 0.01 | 0 |
| **6** | 0 | 0 | 0.2 | 0 | 0.04 | **0.66** | 0.1 | 0 |
| **7** | 0 | 0 | 0.09 | 0 | 0 | 0.01 | **0.85** | 0.05 |
| **8** | 0 | 0 | 0 | 0 | 0 | 0 | 0.11 | **0.89** |

**Table S3. Baseline characteristics by creatinine trajectory in MIMIC-IV**

|  | **Class 1** | **Class 2** | **Class 3** | **Class 4** | **Class 5** | **Class 6** | **Class 7** | **Class 8** | **p-value** |
| --- | --- | --- | --- | --- | --- | --- | --- | --- | --- |
| **N** | **988** | **399** | **715** | **447** | **923** | **135** | **474** | **116** |  |
| **Age (years)** | 72  (17, 63) | 67 (23, 55) | 71 (22, 60) | 67 (22, 56) | 71 (20, 61) | 72 (23, 61) | 67 (21, 56) | 63 (21, 53) | <0.001 |
| **Male (%)** | 643 (65%) | 264 (66%) | 409 (57%) | 284 (64%) | 568 (62%) | 77 (57%) | 266 (56%) | 66 (57%) | 0.002 |
| **Race (%)** |  |  |  |  |  |  |  |  | <0.001 |
| White | 719 (73%) | 248 (62%) | 485 (68%) | 307 (69%) | 640  (70%) | 96 (71%) | 308 (65%) | 80 (69%) |  |
| Black | 74 (7%) | 28 (7%) | 77 (11%) | 43 (10%) | 89 (10%) | 8 (6%) | 43 (9%) | 13 (11%) |  |
| Hispanic | 25 (3%) | 14 (4%) | 25 (3%) | 23 (5%) | 35 (3%) | 3 (2%) | 10 (2%) | 4 (4%) |  |
| Others | 170 (17%) | 109 (27%) | 128 (18%) | 74 (16%) | 159 (17%) | 28 (21%) | 113 (24%) | 19 (16%) |  |
| **Height (cm)** | 170 (163, 178) | 170 (163, 178) | 168 (160, 178) | 173 (163, 178) | 170 (160, 178) | 168 (163, 178) | 168 (160, 178) | 173 (160, 178) | 0.007 |
| **Weight (kg)** | 78.9 (24.2, 68) | 77.1 (26, 65) | 80.6 (29, 68) | 83.9 (30, 69) | 80.4 (28, 68) | 80 (27, 69) | 84.6 (30, 70) | 80.8 (28, 70.7) | 0.001 |
| **Underlying diseases (%)** |  |  |  |  |  |  |  |  |  |
| Diabetes mellitus | 192 (19%) | 51 (13%) | 197 (28%) | 128 (29%) | 221 (24%) | 31 (23%) | 115 (24%) | 16 (14%) | 0.004 |
| Congestive heart failure | 170 (17%) | 56 (14%) | 212 (30%) | 136 (30%) | 231 (25%) | 41 (30%) | 104 (22%) | 18 (16%) | <0.001 |
| Arrhythmia | 136 (14%) | 49 (12%) | 149 (21%) | 101 (23%) | 196 (21%) | 38 (28%) | 87 (18%) | 18 (16%) | <0.001 |
| Chronic lung disease | 118 (12%) | 42 (11%) | 130 (18%) | 75 (17%) | 149 (16%) | 29 (21%) | 68 (14%) | 9 (8%) | <0.001 |
| Liver disease | 48 (5%) | 13 (3%) | 74 (10%) | 51 (11%) | 73 (8%) | 14 (10%) | 45 (9%) | 10 (9%) | <0.001 |
| **Laboratory** |  |  |  |  |  |  |  |  |  |
| Hemoglobin (g/dL) | 8.5 (7.6, 9.6) | 8.5 (7.7, 9.9) | 8.4 (7.3, 9.5) | 8.7 (7.4, 10) | 8.4 (7.5, 9.5) | 8.4 (7.3, 9.2) | 8.3 (7.3, 9.6) | 8.2 (7.3, 9.4) | 0.009 |
| Hematocrit (%) | 25.3 (22.8, 28.6) | 25.3 (22.9, 30) | 25.2 (22.2, 29.3) | 26.3 (22.9, 30.7) | 25 (22.6, 29) | 25.4 (22.6, 28.3) | 25.1 (22.4, 29.2) | 24.9 (22.3, 29) | 0.02 |
| White blood cell count (× 10^9^/L) | 14.8 (11.6, 18.9) | 14.9 (11.5, 19.7) | 15.6 (11.2, 21.7) | 16.3 (11.7, 21.4) | 15.2 (11.4, 20.2) | 16.2 (12.7, 22.4) | 16.5 (11.7, 21.7) | 15.6 (11.6, 21.4) | 0.02 |
| Platelet (x 1000/mm^3^) | 118 (89, 160) | 129 (94, 182) | 128 (79, 187) | 130 (81, 193) | 115 (80, 169) | 103 (61, 180) | 129 (74, 206) | 145 (84, 218) | <0.001 |
| PT | 16 (14, 18) | 16 (14, 18) | 16 (14, 21) | 16 (14, 21) | 16 (14, 20) | 17 (14, 22) | 17 (14, 24) | 16 (14, 27) | <0.001 |
| PTT | 36 (31, 47) | 35 (31, 46) | 38 (31, 63) | 36 (30, 56) | 38 (32, 57) | 42 (33, 72) | 41 (32, 71) | 38 (31, 55) | <0.001 |
| INR | 1.4 (1.3, 1.7) | 1.4 (1.2, 1.7) | 1.5 (1.3, 2) | 1.5 (1.3, 1.9) | 1.5 (1.3, 1.9) | 1.6 (1.3, 2) | 1.6 (1.3, 2.3) | 1.5 (1.3, 2.5) | <0.001 |
| BUN (mg/dL) | 29 (23, 39) | 23 (18, 31) | 50 (35, 69) | 34 (24, 48) | 38 (28, 52) | 53 (40, 68) | 62 (44, 81) | 88 (67, 109) | <0.001 |
| Sodium (mmo/L) | 141 (139, 143) | 141 (139, 144) | 142 (140, 146) | 142 (140, 145) | 142 (139, 145) | 141 (138, 144) | 143 (140, 146) | 143 (139, 146) | <0.001 |
| Potassium (mmo/L) | 4.7 (4.4, 5) | 4.6 (4.3, 5) | 4.8 (4.4, 5.3) | 4.7 (4.3, 5.2) | 4.8 (4.5, 5.2) | 4.7 (4.4, 5.2) | 4.9 (4.4, 5.4) | 5.2 (4.5, 5.6) | <0.001 |
| Chloride (mmo/L) | 110 (108, 113) | 109 (106, 114) | 109 (105, 113) | 108 (105, 112) | 109 (106, 112) | 107 (104, 111) | 109 (105, 113) | 107 (103, 112) | <0.001 |
| Bicarbonate (mmo/L) | 22 (19, 23) | 21 (18, 23) | 19 (16, 22) | 20 (17, 23) | 21 (18, 23) | 19 (18, 23) | 17 (14, 20) | 16 (13, 18) | <0.001 |
| Calcium (mg/dL) | 8.6 (8.2, 8.9) | 8.6 (8.2, 8.9) | 8.6 (8.3, 9) | 8.7 (8.3, 9.2) | 8.7 (8.3, 9.1) | 8.6 (8.2, 9.2) | 8.6 (8.2, 9.1) | 8.6 (8.2, 9) | <0.001 |
| SGOT (U/L) | 45 (27, 99) | 50 (28, 117) | 54 (29, 135) | 56 (28, 148) | 51 (29, 130) | 85 (35, 301) | 73 (33, 301) | 80 (31, 374 | <0.001 |
| SGPT (U/L) | 30 (17, 71) | 37 (18, 112) | 36 (19, 102) | 38 (20, 129) | 35 (18, 88) | 39 (21, 166) | 43 (19, 177) | 35 (19, 212) | <0.001 |
| ALP (U/L) | 75 (54, 107) | 81 (60, 125) | 95 (68, 138) | 88 (64, 129) | 83 (60, 122) | 88 (61, 124) | 101 (70, 167) | 107 (75, 158) | <0.001 |
| Albumin (d/dL) | 3 (2.6, 3.3) | 2.8 (2.4, 3.2) | 2.8 (2.4, 3.2) | 2.9 (2.4, 3.3) | 2.9 (2.5, 3.3) | 2.7 (2.3, 3.2) | 2.7 (2.2, 3.1) | 2.7 (2.3, 3.1) | <0.001 |
| Glucose (mg/dL) | 157 (133, 197) | 164 (138, 210) | 187 (147, 245) | 178 (148, 249) | 173 (142, 228) | 169 (141, 236) | 184 (146, 249) | 176 (251, 143) | <0.001 |
| pH | 7.32 (7.27, 7.36) | 7.32 (7.26, 7.37) | 7.29 (7.22, 7.35) | 7.3 (7.24, 7.36) | 7.3 (7.24, 7.35) | 7.26 (7.19, 7.32) | 7.27 (7.19, 7.33) | 7.28 (7.2, 7.34) | <0.001 |
| pO2 | 78 (52, 103) | 72 (43, 102) | 53 (37, 75) | 55 (36, 82) | 63 (41, 88) | 54 (40, 72) | 52 (38, 75) | 49 (37, 75) | <0.001 |
| pCO2 | 47 (43, 53) | 46 (42, 53) | 48 (40, 56) | 48 (41, 57) | 48 (42, 56) | 49 (45, 59) | 46 (39, 54) | 43 (36, 50) | <0.001 |
| pO2/FiO2 | 332 (260, 420) | 326 (247, 431) | 307 (226, 397) | 300 (208, 400) | 318 (234, 410) | 306 (238, 412) | 319 (223, 420) | 314 (236, 428) | <0.001 |
| Lactate (mg/dL) | 2.4 (1.7, 3.3) | 2.3 (1.7, 3.4) | 2.5 (1.6, 3.9) | 2.5 (1.7, 4) | 2.5 (1.8, 3.7) | 2.9 (1.8, 4.8) | 2.4 (1.6, 4.2) | 2.1 (1.5, 3.5) | 0.009 |
| **Baseline creatinine (mg/dL)** | 1 (0.9, 1.3 | 1.1 (0.9, 1.3) | 1 (0.8, 1.1) | 0.9 (0.7, 1.1) | 1 (0.8, 1.4) | 1.1 (0.9, 1.4) | 0.9 (0.8, 1.1) | 0.8 (0.7, 1) | <0.001 |
| **First AKI staging (%)** |  |  |  |  |  |  |  |  | <0.001 |
| 1 | 985 (99.7%) | 395 (99%) | 313 (44%) | 397 (89%) | 896 (97%) | 132 (97.9%) | 102 (22%) | 8 (7%) |  |
| 2 | 1 (0.1%) | 0 | 349 (49%) | 36 (8%) | 12 (1%) | 1 (0.7) | 149 (31%) | 8 (7%) |  |
| 3 | 2 (0.2%) | 4 (1%) | 53 (7%) | 14 (3%) | 15 (2%)) | 2 (1.4%) | 223 (47%) | 100 (86%) |  |
| **MAX AKI Staging (%)** |  |  |  |  |  |  |  |  | <0.001 |
| 1 | 969 (98.1%) | 390 (98%) | 193 (13%) | 389 (87%) | 808 (88%) | 4 (3%) | 2 (0.4%) | 0 |  |
| 2 | 1 (0.1%) | 0 | 430 (74%) | 44 (10%) | 67 (7%) | 78 (57%) | 46 (9.6%) | 1 (1%) |  |
| 3 | 18 (1.8%) | 9 (2%) | 92 (13%) | 14 (3%) | 48 (5%) | 53 (40%) | 426 (90%) | 115 (99%) |  |
| **MAX SOFA score** | 6 (5, 8) | 6 (4, 8) | 8 (6, 11) | 7 (5, 10) | 7 (5, 10) | 10 (7, 13) | 9 (7, 12) | 9 (7, 12 ) | <0.001 |
| **Vasopressor uses (%)** | 658 (67%) | 249 (62%) | 421 (59%) | 243 (54%) | 558 (60%) | 96 (71%) | 312 (66%) | 66 (57%) | <0.001 |
| **Vasopressor duration (minutes)** | 740 (184, 1876) | 1001 (285, 2595) | 2204 (834, 4119) | 1526 (695, 3018) | 1080 (396, 2683) | 2419 (568, 3018) | 2347 (997, 4032) | 2326 (781, 3919) | <0.001 |
| **Nephrotoxins (%)** | 303 (31%) | 125 (31%) | 271 (38%) | 177 (40%) | 309 (33%) | 46 (34%) | 163 (34%) | 47 (41%) | 0.007 |

Data are presented as count (percent) or median (interquartile range [IQR])

Abbreviation: AKI- acute kidney injury; BUN- blood urea nitrogen; SGPT- serum glutamic pyruvic transaminase; SGOT- serum glutamic-oxaloacetic transaminase; SOFA- sequential Organ Failure Assessment

**Table S4** Clinical outcomes by creatinine trajectory in MIMIC-IV

|  | **Class 1** | **Class 2** | **Class 3** | **Class 4** | **Class 5** | **Class 6** | **Class 7** | **Class 8** | **P value** |
| --- | --- | --- | --- | --- | --- | --- | --- | --- | --- |
| N | 988 | 399 | 715 | 447 | 923 | 135 | 474 | 116 |  |
| AKD | 44(4.5%) | 24(6%) | 177(24.8%) | 39(8.7%) | 142(15.4%) | 58(43%) | 146(30.8%) | 44(37.9%) | < 0.001 |
| AKD + death day7 | 67(6.8%) | 41(10.3%) | 213(29.8%) | 45(10.1%) | 185(20%) | 81(60%) | 186(39.2%) | 54(46.6%) | < 0.001 |
| AKD + death by discharge | 94(9.5%) | 59(14.8%) | 172(24.1%) | 38(8.5%) | 165(17.9%) | 72(53.3%) | 135(28.5%) | 37(31.9%) | < 0.001 |

Data are presented as count (percent)

Abbreviation: AKD- acute kidney disease

**Table S5. Mean Posterior Probability of Membership in Class in eICU database**

| **Class** | **1** | **2** | **3** | **4** | **5** | **6** | **7** | **8** |
| --- | --- | --- | --- | --- | --- | --- | --- | --- |
| **1** | **0.61** | 0.12 | 0.02 | 0.04 | 0.21 | 0 | 0 | 0 |
| **2** | 0.12 | **0.76** | 0.03 | 0.02 | 0.06 | 0 | 0.01 | 0 |
| **3** | 0 | 0 | **0.72** | 0.06 | 0.06 | 0.02 | 0.14 | 0 |
| **4** | 0.02 | 0.01 | 0.19 | **0.60** | 0.17 | 0 | 0.01 | 0 |
| **5** | 0.07 | 0 | 0.15 | 0.14 | **0.62** | 0.01 | 0.01 | 0 |
| **6** | 0 | 0 | 0.23 | 0 | 0.03 | **0.61** | 0.13 | 0 |
| **7** | 0 | 0 | 0.07 | 0 | 0 | 0.01 | **0.86** | 0.06 |
| **8** | 0 | 0 | 0 | 0 | 0 | 0 | 0.09 | **0.91** |

**Table S6. Baseline characteristics by creatinine trajectory in eICU database**

|  | **Class 1** | **Class 2** | **Class 3** | **Class 4** | **Class 5** | **Class 6** | **Class 7** | **Class 8** | **p-value** |
| --- | --- | --- | --- | --- | --- | --- | --- | --- | --- |
| **N** | **192** | **73** | **1275** | **471** | **588** | **71** | **1019** | **274** |  |
| **Age (years)** | 66 (57, 76) | 58 (42, 65) | 72 (62, 81) | 68 (59, 77) | 72 (61, 80) | 70 (56, 78) | 67 (57, 78) | 62 (53, 71) | <0.001 |
| **Male (%)** | 139 (72%) | 54 (74%) | 688 (54%) | 282 (60%) | 358 (61%) | 42 (59%) | 534 (52%) | 131 (48%) | <0.001 |
| **Race (%)** |  |  |  |  |  |  |  |  | 0.003 |
| White | 160 (83%) | 60 (82%) | 1036 (81%) | 384 (82%) | 482 (82%) | 53 (75%) | 798 (78%) | 198 (72%) |  |
| Black | 12 (6%) | 5 (7%) | 151 (12%) | 46 (10%) | 46 (8%) | 7 (10%) | 128 (13%) | 39 (14%) |  |
| Hispanic | 5 (3%) | 2 (3%) | 13 (1%) | 10 (2%) | 9 (2%) | 1 (1%) | 19 (2%) | 5 (2%) |  |
| Others | 15 (8%) | 6 (8%) | 75 (6%) | 31 (6%) | 51 (8%) | 10 (14%) | 74 (7%) | 32 (12%) |  |
| **Height (cm)** | 173 (165,178) | 175 (165, 180) | 168 (163, 178) | 170 (163, 178) | 170 (163, 178) | 170 (161, 178) | 170 (163, 178) | 168 (163, 178) | 0.006 |
| **Weight (kg)** | 89 (71, 103) | 86 (67, 106) | 86 (71, 104) | 81.6 (69, 100) | 83 (69, 102) | 77 (62, 96) | 85 (70, 105) | 88.4 (71, 108) | 0.004 |
| **Underlying diseases (%)** |  |  |  |  |  |  |  |  |  |
| Diabetes mellitus | 13 (7%) | 11 (15%) | 179 (14%) | 43 (9%) | 64 (11%) | 7 (10%) | 126 (12%) | 24 (9%) | 0.01 |
| Congestive heart failure | 18 (10%) | 3 (4%) | 232 (18%) | 47 (10%) | 90 (15%) | 12 (17%) | 137 (13%) | 21 (8%) | <0.001 |
| Arrhythmia | 37 (19%) | 6 (8%) | 202 (16%) | 74 (16%) | 79 (13%) | 17 (24%) | 145 (14%) | 27 (10%) | 0.01 |
| Chronic lung disease | 16 (8%) | 4 (5%) | 161 (13%) | 51 (11%) | 56 (10%) | 7 (10%) | 76 (7%) | 8 (3%) | <0.001 |
| Liver disease | 7 (4%) | 2 (3%) | 62 (5%) | 23 (5%) | 18 (3%) | 7 (10%) | 50 (5%) | 11 (4%) | 0.22 |
| **Laboratory** |  |  |  |  |  |  |  |  |  |
| Hemoglobin (g/dL) | 8.8 (8, 10) | 9.4 (7.9, 10.6) | 8.8 (7.6, 10.3) | 9.3 (7.9, 10.7) | 8.7 (7.7, 10.2) | 8.6 (7.3, 9.85) | 8.6 (7.5, 10) | 8.5 (7.4, 9.7) | <0.001 |
| Hematocrit (%) | 26.5 (23.5, 30.8) | 27.8 (23.7, 31.9) | 27.2 (23.5, 31.6) | 28.5 (24.4, 33) | 26.6 (23.4, 31) | 25 (22.9, 29.9) | 26.5 (22.9, 30.5) | 26 (22.4, 29.2) | <0.001 |
| White blood cell count (× 10^9^/L) | 15.6 (11.6, 19.4) | 14.6 (11.7, 19.4) | 14.9 (10.9, 21) | 14.9 (10.8, 19.7) | 15.5 (11.8, 20.1) | 18.3 (13.1, 22.5) | 16.4 (11.7, 22) | 13.8 (10.2, 20.5) | <0.001 |
| Platelet (x 1000/mm^3^) | 110 (80, 158) | 140 (111, 180) | 132 (88, 187) | 133 (93, 192) | 122 (87, 181) | 112 (65, 190) | 126 (76, 182) | 141 (91, 192) | <0.001 |
| PT | 17 (15, 20) | 16 (14, 19) | 18 (15, 24) | 16 (14, 22) | 17 (14, 20) | 17 (14, 25) | 18 (15, 24) | 16 (14, 20) | <0.001 |
| PTT | 37 (30, 49) | 36 (31, 62 | 39 (32, 55) | 38 (61, 55) | 37 (31, 54 | 53 (37, 86) | 39 (32, 57) | 36 (31, 51) | <0.001 |
| INR | 1.4 (1.2, 1.8) | 1.4 (1.2, 1.6) | 1.5 (1.2, 2.2) | 1.4 (1.2, 1.9) | 1.4 (1.2, 1.8) | 1.4 (1.2, 2.3) | 1.5 (1.2, 2.1) | 1.4 (1.2, 1.8) | <0.001 |
| BUN (mg/dL) | 27 (22, 34) | 21 (16, 27) | 46 (34, 58) | 31 (25, 42) | 34 (26, 43) | 46 (38, 58) | 60 (45, 77) | 71 (51, 92) | <0.001 |
| Sodium (mmo/L) | 142 (140, 144) | 144 (141, 147) | 143 (140, 146) | 143 (140, 147) | 143 (140, 146) | 143 (139, 147) | 142 (139, 146) | 141 (138, 145) | <0.001 |
| Potassium (mmo/L) | 4.6 (4.2, 4.9) | 4.4 (4.2, 4.8) | 4.6 (4.2, 5.1) | 4.5 (4.1, 4.9) | 4.6 (4.3, 5.1) | 4.7 (4.4, 5.1) | 4.8 (4.3, 5.4) | 5 (4.4, 5.6) | <0.001 |
| Chloride (mmo/L) | 110 (107, 113 | 111 (107, 116) | 110 (105, 114) | 110 (107, 114) | 109 (105, 113) | 110 (107, 114) | 109 (104, 113) | 105 (100, 111) | <0.001 |
| Bicarbonate (mmo/L) | 21 (19, 23) | 21 (17, 24 | 20 (16, 23) | 20 (17, 23) | 21 (18, 23) | 18 (15, 22 ) | 18 (15, 21) | 18 (14, 22) | <0.001 |
| Calcium (mg/dL) | 8.7 (8.3, 9.1) | 8.5 (8.3, 8.9) | 8.6 (8.1, 9) | 8.5 (8.1, 8.9) | 8.7 (8.2, 9) | 8.5 (8.1, 8.8) | 8.5 (8, 9) | 8.5 (7.9, 9.1) | <0.001 |
| SGOT (U/L) | 42 (26, 88) | 40 (24, 87) | 41 (24, 102) | 46 (24, 114) | 41 (25, 86) | 62 (34, 151) | 53 (27, 187) | 49 (25, 148) | <0.001 |
| SGPT (U/L) | 28 (17, 50) | 31 (19, 59) | 30 (17, 70) | 31 (20, 79) | 29 (18, 63) | 38 (22, 81) | 34 (19, 120) | 33 (19, 105) | <0.001 |
| ALP (U/L) | 67 (50, 92) | 75 (55, 102) | 84 (61, 124) | 78 (58, 114) | 77 (56, 104) | 89 (70, 116) | 94 (67, 144) | 97 (73, 151) | <0.001 |
| Albumin (d/dL) | 2.8 (2.3, 3.2) | 2.6 (2.1, 3) | 2.5 (2.1 ,2.9) | 2.5 (2.1, 2.9) | 2.6 (2.2, 3.1) | 2.4 (2, 2.9) | 2.4 (2, 2.8) | 2.5 (2, 2.9) | <0.001 |
| Glucose (mg/dL) | 165 (142, 206) | 188 (156, 232) | 185 (147, 250) | 177 (144, 236) | 180 (150, 237) | 183 (161, 221) | 189 (148, 262) | 179 (145, 233) | <0.001 |
| pH | 7.33 (7.29, 7.38) | 7.33 (7.27, 7.41) | 7.32 (7.24, 7.38) | 7.33 (7.26, 7.38) | 7.32 (7.26, 7.38) | 7.29 (7.2, 7.37) | 7.29 (7.22, 7.35) | 7.29 (7.2, 7.36) | <0.001 |
| pO2 | 76 (63, 98) | 76 (63, 108) | 75 (63, 94) | 76 (63, 102) | 74 (62, 92) | 66 (54, 89) | 75 (61, 96) | 73 (60, 91) | 0.08 |
| pCO2 | 44 (40, 49) | 44 (37, 50) | 43 (37, 52) | 43 (37, 51) | 45 (39, 54) | 45 (38, 56) | 42 (36, 51) | 43 (37, 50) | <0.001 |
| pO2/FiO2 | 278 (241, 371) | 323 (238, 402) | 285 (214, 370) | 295 (218, 374) | 286 (210, 367) | 272 (196, 376) | 293 (218, 385) | 300 (220, 384) | 0.24 |
| Lactate (mg/dL) | 1.8 (1.3, 3) | 2.1 (1.4, 3.5) | 2.1 (1.3, 3.5) | 2 (1.8, 3.8) | 2 (1.3, 3.5) | 2 (1.4, 4) | 2.1 (1.3, 4) | 1.7 (1.1, 2.8) | <0.001 |
| **Baseline creatinine (mg/dL)** | 1 (0.8, 1) | 1 (0.8, 1.1) | 1 (0.8, 1) | 0.96 (0.8, 1) | 0.9 (0.8, 1) | 0.9 (0.8, 1) | 1 (0.8, 1) | 0.9 (0.8, 1.1) | <0.001 |
| **First AKI staging (%)** |  |  |  |  |  |  |  |  | <0.001 |
| 1 | 192 (100%) | 71 (97%) | 550 (43%) | 449 (95.3%) | 576 (98%) | 65 (92%) | 136 (13%) | 8 (3%) |  |
| 2 | 0 | 0 | 715 (56%) | 21 (4.5%) | 11 (1.8%) | 5 (7%) | 342 (34%) | 20 (7%) |  |
| 3 | 0 | 2 (3%) | 10 (1%) | 1 (0.2%) | 1 (0.2%) | 1 (1%) | 541 (53%) | 246 (90%) |  |
| **MAX AKI Staging (%)** |  |  |  |  |  |  |  |  | <0.001 |
| 1 | 192 (100%) | 69 (95%) | 246 (19%) | 443 (94%) | 539 (91%) | 5 (7%) | 14 (1.5%) | 0 |  |
| 2 | 0 | 0 | 992 (78%) | 27 (5.8%) | 45 (8%) | 53 (75%) | 87 (8.5%) | 0 |  |
| 3 | 0 | 4 (5%) | 37 (3%) | 1 (0.2%) | 4 (1%) | 13 (18%) | 918 (90%) | 274 (100%) |  |
| **MAX SOFA score** | 7 (5, 9) | 7 (5, 9) | 7 (5, 10) | 7 (5, 9) | 7 (5, 10) | 10 (7, 12) | 9 (6, 12) | 9 (6, 11) | <0.001 |
| **Vasopressor uses (%)** | 42 (22%) | 21 (29%) | 316 (25%) | 108 (23%) | 151 (26%) | 26 (37%) | 286 (28%) | 69 (25%) | 0.11 |
| **Vasopressor duration (minutes)** | 2805 (840, 5018) | 2235 (840, 4934) | 2680 (960, 4853) | 2420 (840, 4682) | 2222 (840, 4682) | 2901 (1342, 4539) | 3062 (1055, 5145) | 3140 (1380, 4766) | <0.001 |
| **Nephrotoxins (%)** | 144 (75%) | 54 (74%) | 944 (74%) | 355 (75%) | 454 (77%) | 58 (82%) | 756 (74%) | 181 (66%) | 0.04 |

Data are presented as count (percent) or median (interquartile range [IQR])

Abbreviation: AKI- acute kidney injury; BUN- blood urea nitrogen; SGPT- serum glutamic pyruvic transaminase; SGOT- serum glutamic-oxaloacetic transaminase; SOFA- sequential Organ Failure Assessment

**Table S7** Clinical outcomes by creatinine trajectory in eICU database

|  | **Class 1** | **Class 2** | **Class 3** | **Class 4** | **Class 5** | **Class 6** | **Class 7** | **Class 8** | **P value** |
| --- | --- | --- | --- | --- | --- | --- | --- | --- | --- |
| N | 192 | 73 | 1275 | 471 | 588 | 71 | 1019 | 274 |  |
| AKD | 6(3.1%) | 1(1.4%) | 319(25%) | 41(8.7%) | 82(13.9%) | 29(40.8%) | 404(39.6%) | 118(43.1%) | <0.001 |
| AKD + death day7 | 11(5.7%) | 6(8.2%) | 370(29%) | 53(11.3%) | 98(16.7%) | 37(52.1%) | 472(46.3%) | 137(50%) | <0.001 |
| AKD + death by discharge | 12(6.3%) | 7(9.6%) | 352(27.6%) | 41(8.7%) | 106(18%) | 33(46.5%) | 339(33.3%) | 118(43.1%) | <0.001 |

Data are presented as count (percent)

Abbreviation: AKD- acute kidney disease
